# Quarantine and serial testing for variants of SARS-CoV-2 with benefits of vaccination and boosting on consequent control of COVID-19

**DOI:** 10.1093/pnasnexus/pgac100

**Published:** 2022-07-27

**Authors:** Chad R Wells, Abhishek Pandey, Senay Gokcebel, Gary Krieger, A Michael Donoghue, Burton H Singer, Seyed M Moghadas, Alison P Galvani, Jeffrey P Townsend

**Affiliations:** Center for Infectious Disease Modeling and Analysis (CIDMA), Yale School of Public Health, New Haven, CT 06520, USA; Center for Infectious Disease Modeling and Analysis (CIDMA), Yale School of Public Health, New Haven, CT 06520, USA; Department of Biostatistics, Yale School of Public Health, New Haven, CT 06510, USA; Department of Biochemistry, Grinnell College, Grinnell, IA 50112, USA; NewFields E&E, Boulder, CO 80301, USA; Skaggs School of Pharmacy and Pharmaceutical Science, University of Colorado Anschutz Medical Campus, Aurora, CO 80045, USA; Group HSE, BHP Group Ltd, 171 Collins Street, Melbourne, VIC 3000, Australia; Emerging Pathogens Institute, University of Florida, P.O. Box 100009, Gainesville, FL 32610, USA; Agent-Based Modelling Laboratory, York University, Toronto, ON M3J 1P3, Canada; Center for Infectious Disease Modeling and Analysis (CIDMA), Yale School of Public Health, New Haven, CT 06520, USA; Department of Biostatistics, Yale School of Public Health, New Haven, CT 06510, USA; Program in Computational Biology and Bioinformatics, Yale University, New Haven, CT 06511, USA; Program in Microbiology, Yale University, New Haven, CT 06511, USA

**Keywords:** severe acute respiratory syndrome, variants of concern, public health, disease time course, omicron

## Abstract

Quarantine and serial testing strategies for a disease depend principally on its incubation period and infectiousness profile. In the context of COVID-19, these primary public health tools must be modulated with successive SARS CoV-2 variants of concern that dominate transmission. Our analysis shows that (1) vaccination status of an individual makes little difference to the determination of the appropriate quarantine duration of an infected case, whereas vaccination coverage of the population can have a substantial effect on this duration, (2) successive variants can challenge disease control efforts by their earlier and increased transmission in the disease time course relative to prior variants, and (3) sufficient vaccine boosting of a population substantially aids the suppression of local transmission through frequent serial testing. For instance, with Omicron, increasing immunity through vaccination and boosters—for instance with 100% of the population is fully immunized and at least 24% having received a third dose—can reduce quarantine durations by up to 2 d, as well as substantially aid in the repression of outbreaks through serial testing. Our analysis highlights the paramount importance of maintaining high population immunity, preferably by booster uptake, and the role of quarantine and testing to control the spread of SARS CoV-2.

Significance Statement Serial emergent SARS CoV-2 variants with divergent properties have dominated transmission over the pandemic, even as vaccination and boosting have affected risks. Quarantine and serial testing strategies should be adjusted to account for these factors. For example, successive variants have exhibited earlier and greater transmission compared to prior variants, limiting the effectiveness of some disease control efforts; and up-to-date vaccination and boosting continue to lower the probability of contracting disease. Using a data-driven model to quantify onward transmission, we found that population-level vaccination coverage impacts quarantine duration. Moreover, increasing booster coverage substantially enhances the effects of serial testing on reducing community transmission. High population immunity through vaccination and boosting substantially aids the prevention of transmission via quarantine and testing.

## Introduction

Quarantine and testing have been essential public health tools to curb the spread of SARS CoV-2 (1–4). The successive emergence and dominance of variants of concern (VOC)—each with distinct clinical traits and transmissibility—have challenged these control efforts, even with the aid of vaccination (5, 6). Consequently, the effectiveness and practicality of quarantine and serial testing in a population are subject to change. Initial quarantines for close contacts of a COVID-19 case lasted 14 d, later were shortened to seven, and then 5 d (7, 8). Recently, some countries have stipulated quarantine durations dependent on the vaccine status of the individual (7, 9). Some organizations have further conducted serial testing (as part of screening programs) to mitigate outbreaks in their populations (10, 11), while adjusting the testing frequency based on community prevalence or the incubation period (12, 13). These policy directives respond to a widespread perception that public health interventions require adaptation to increasing population immunity and virus evolution. However, the appropriate quarantine and testing policies for diverse vaccination levels in the face of successive VOCs have not been evaluated to gauge changing policy effectiveness for mitigating transmission.

Here we adapt previous approaches (1–3) to compare the probability of post-quarantine transmission (PQT) and the effective reproduction number with serial testing and isolation for the original SARS CoV-2 strain and three VOCs—Alpha, Delta, and Omicron—at different levels of vaccination. Our findings demonstrate that quarantine and testing strategies depend not only on the incubation period but also on the transmissibility and level of population immunity against infection.

## Results

**Fig. 1.**
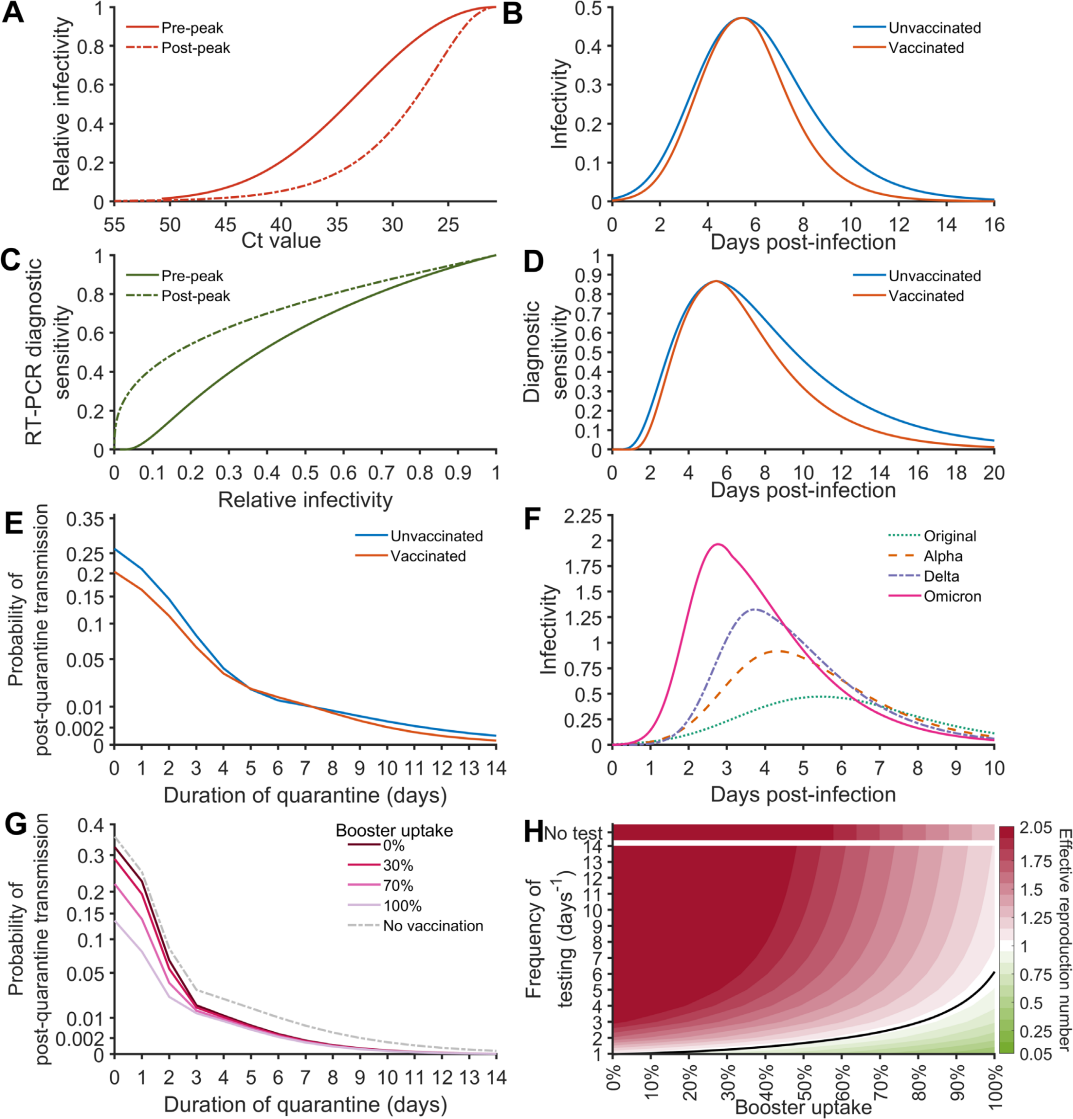
Influence of SARS CoV-2 infection characteristics on disease control efforts. The (**A**) relationship between the *Ct* value and relative infectivity for the time prior to peak infectivity (solid line) and the time following peak infectivity (dashed line) for which *Ct* values greater than 40 were determined from a linear extrapolation, (**B**) infectivity over the disease time course, (**C**) the relationship between the relative infectivity and the RT-PCR diagnostic sensitivity for the time prior to peak infectivity (solid line) and the time following peak infectivity (dashed line), (**D**) temporal rapid antigen test diagnostic sensitivity, and (**E**) probability of post-quarantine transmission for an individual infected with an original strain of SARS-CoV-2 who has not been vaccinated (blue) and who has received two vaccine doses (orange). The (**F**) infectivity over the disease time course for original SARS-CoV-2 strain (green dotted) and the Alpha (orange dashed), Delta (purple dash dot), and Omicron (pink solid) variants. For a case infected by Omicron with no vaccination (gray dashed), 100% uptake of two vaccine doses and 0%, 30%, 70%, and 100% boosting, the (**G**) probability of post-quarantine transmission for quarantine durations from 0 to 14 d with a rapid antigen test on exit, and (**H**) the effective reproduction number (*R_E_* > 1: red, *R_E_* < 1: green, and *R_E_* = 1: black line) for no testing and for testing frequencies from every day to every 14 d.

Using the specified infectivity profile, the temporal diagnostic sensitivity of a rapid antigen test, and proportion of cases self-isolating due to the appearance of symptoms, we computed the number of secondary infections. The comparison of the number of post-quarantine secondary infections between unvaccinated and vaccinated individuals demonstrates that the vaccination status of an individual has only a limited effect on quarantine duration. A shorter duration of viral shedding in breakthrough infections after two vaccine doses compared to primary infection in unvaccinated individuals (14) leads to fewer secondary infections (Fig. 1B), but simultaneously decreases the probability of detecting a case (Fig. 1D). Furthermore, vaccinated cases are less likely to exhibit symptoms than unvaccinated, resulting in less frequent self-isolation. These decreases in case detection and self-isolation increase the probability of releasing an infectious case from quarantine. The tradeoff between lower infectivity and higher risk of release leads to marginal differences in PQT between unvaccinated and vaccinated cases (Fig. 1E).

Successively predominant VOCs analyzed in this study exhibited consecutively shorter incubation periods and increasing transmission rates. These two characteristics contribute to a greater risk of transmission earlier in the disease time course than the original SARS CoV-2 strain (Fig. 1F), making effective outbreak suppression through serial testing challenging.

**Fig. 2.**
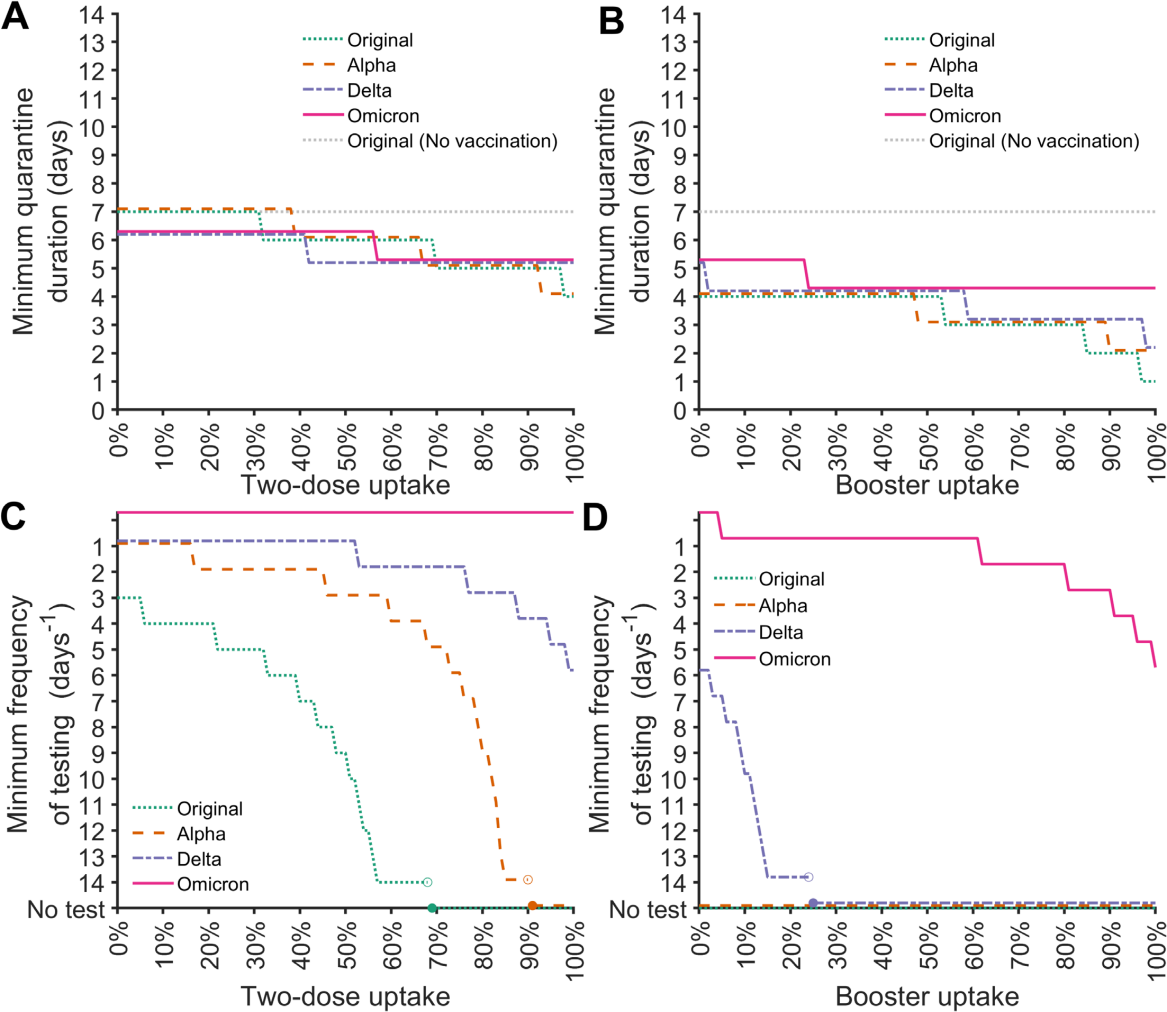
Optimal quarantine and serial testing strategies using a rapid antigen test for four SARS CoV-2 variants. The minimum quarantine duration with a rapid antigen test on exit that provides equivalent or lower probability of post-quarantine transmission than a 7-d quarantine with a rapid antigen test for the original strain in an immunologically naive population (gray dotted), for (**A**) up to 100% two-dose uptake, and for (**B**) 100% two-dose uptake plus 0% to 100% booster uptake for the original strain(s) (green dotted), as well as Alpha (orange dashed), Delta (purple dash–dotted), and Omicron variants of concern (pink solid). The minimum frequency of serial rapid antigen testing to obtain an effective reproduction number (*R_E_*) below one with (**C**) 0% to 100% two-dose uptake and (**D**) with 100% two-dose uptake plus 0% to 100% booster uptake. When daily testing was determined to be insufficient to suppress an outbreak (i.e. *R_E_* ≥ 1; only occurring with serial testing for Omicron), the minimum frequency of testing line was plotted level at the top of the *y* axis.

Determining optimal disease control strategies also depends on the level of population immunity. Increasing population immunity through vaccine and booster uptake mitigates transmission (Fig. 1G–H). Specifically, improving two-dose vaccine uptake leads to a substantial decrease in PQT and transmission during serial testing (Fig. 1G). In contrast, increasing booster uptake provides only substantive benefit to serial testing compared to quarantine (Fig. 1G vs. Fig. 1H; Fig. 2). The decrease in PQT concomitant with an increase in booster uptake diminishes as quarantine durations lengthen (Fig. 1G). The effectiveness of serial testing in reducing transmission is greatly improved through an increase in booster uptake (Fig. 1H).

Despite the shorter incubation period of Omicron compared to the previous VOCs, a longer quarantine duration relative to the incubation period could be needed to obtain a probability of PQT that is equal to or lower than a 7-d quarantine plus exit test applied to the original variant in a completely immunologically naive population (Fig. 2A). Scaling up vaccine-acquired immunity from no vaccination to 100% booster uptake would yield a reduction in quarantine by 6 d for the original SARS CoV-2 strain, but only yield a reduction in quarantine by 2 d for the Omicron variant (Fig. 2A–B). In a population with 100% two-dose vaccination, scaling up booster uptake could justify a reduction of quarantine by as much as 3 d (Fig. 2B). However, boosting only has a moderate impact on quarantine duration for Omicron, decreasing it by at most a day (Fig. 2B). In the absence of boosters, even daily serial rapid antigen testing could not reduce the effective reproduction number *R_E_* of Omicron below one (Fig. 2C). In contrast, increasing booster uptake can result in striking differences in the required frequency of serial testing. For instance, increasing booster uptake from 60% to 85% reduces the necessary serial testing frequency from every day to every three days to bring *R_E_* below one for Omicron (Fig. 2D).

## Discussion

Here we have shown that the vaccination status of an infected individual has limited influence on the post-quarantine transmission. VOCs have produced increasing amounts of transmission earlier in the disease time course, challenging the effectiveness of current quarantine and testing strategies in the suppression of transmission. We show that increasing population immunity through vaccination not only decreases transmission, but can also allow for substantial relaxation of requirements for quarantine and testing. For quarantine, increasing uptake of two doses enables notable reductions in quarantine durations. The additional reduction in PQT obtained through boosting was limited—especially for highly transmissible variants such as Omicron. In the context of serial testing for the less transmissible variants, higher population uptake of two doses could justify less frequent surveillance. In contrast, highly transmissible variants like Omicron require frequent testing and a high booster vaccination uptake to effectively reduce community transmission. Therefore, our results demonstrate that despite increasing transmission and immuno-evasion of successive VOCs, high population immunity against infection—preferably conferred through vaccination—can mitigate transmission.

Rapid antigen testing is conducted in many workplaces to identify and isolate infected workers. The testing frequency that is necessary to contain and suppress any workplace transmission is crucial to prevention of large-scale outbreaks. Our results inform the minimum frequency of rapid antigen testing required to contain and suppress workplace transmission for varying degrees of vaccination in relevant workforces. We accounted for isolation of those who test positive, but not for quarantining of close contacts or the use of additional non-pharmaceutical interventions such as mask wearing. Rapid antigen testing requires a frequency of every 6 d for surveillance to curtail transmission within a population that is 100% boosted. The knowledge that the presence of higher proportions of boosted workers enable reduced frequencies of rapid antigen testing should encourage booster uptake.

Some countries have eliminated quarantine for vaccinated/boosted individuals—requiring only testing, strict mask use, and isolation upon symptom onset (7, 9). At the individual level, removing quarantine for vaccinated individuals might seem reasonable because the probability of entering quarantine as a breakthrough case is lower than an unvaccinated case due to the effectiveness of the vaccine in preventing infection. However, removing quarantine for vaccinated individuals is only appropriate to the extent that they are less likely to turn out to be infected; any single breakthrough infection leads to PQT that is comparable to an infection in an unvaccinated individual. Indeed, as vaccine uptake increases, more cases entering quarantine will be breakthrough infections. We have shown that the quarantine duration required to attain a particular level of transmission risk depends on the vaccine-induced immunity in the population into which a quarantined individual is released. Infection-acquired immunity can also influence optimal quarantine and testing strategies. Thus, surveillance of the immunological status of the population is much more important than the status of the quarantined individual when determining effective quarantine durations (1).

After the emergence of Omicron, the US Centers for Disease Control and Prevention shortened their recommended 7-d quarantine of close contacts to 5 d for the unvaccinated and no quarantine for vaccinated (7, 8). This adjusted quarantine policy might not effectively mitigate transmission, in part due to a joint consequence of the immuno-evasive capabilities and high transmissibility of Omicron, the inadequate (∼66%) full immunization in the US, and even lower rate of ∼46% booster uptake of eligible individuals as of 2022 May 8 (15). Increasing immunity through vaccination enables a moderate reduction in quarantine duration for Omicron. Increasing booster uptake, however, was shown to have a more significant effect on reducing Omicron transmission. To lessen the burden on healthcare systems, continued efforts to administer vaccines and booster doses is essential in preventing large outbreaks with emerging VOCs or their subvariants.

## Method

### Infectivity

To construct the infectivity over the course of disease for each variant, we used the approach described by Ferretti et al. (16), as well as variant-specific incubation periods, basic reproduction numbers, and temporal changes in *Ct* values (14, 17) (Supplementary Material). The temporal changes of the *Ct* values for each variant were informed by the linear slopes computed in previous studies (14, 17).

To account for the effect of vaccination and VOCs on infectivity, we constructed a mapping from the *Ct* value to infectivity using the measurements from the original SARS CoV-2 strain (Fig. 1A; Supplementary Material).

### Rapid antigen test diagnostic sensitivity

The diagnostic sensitivity of the rapid antigen test (Abbott Panbio) is the product of the % positive agreement (PPA) and RT-PCR diagnostic sensitivity for the specified time post-infection (1, 3). To determine the temporal RT-PCR diagnostic sensitivity for each VOC, we constructed a mapping between the relative infectivity and RT-PCR diagnostic sensitivity (Fig. 1C; Supplementary Material). Similarly, the temporal PPA for each VOC was constructed using a mapping between the relative infectivity and PPA.

To calculate the temporal RT-PCR diagnostic sensitivity for our benchmark incubation period, we fitted a log-Normal distribution to serial testing data from Hellewell et al. (18) using a maximum-likelihood approach (3). For our benchmark PPA curve, a linear logit model was fitted to data of the PPA between Abbott Panbio and RT-PCR tests post-symptom onset (19, 20) using a maximum-likelihood approach (1, 3). The studies providing the PPA for the Abbott Panbio rapid antigen test were conducted in community testing centers in the Netherlands and in Aruba (19), as well as in healthcare settings in Madrid (20).

*Post-quarantine transmission* (PQT)

To calculate the PQT for random entry into quarantine and rapid antigen test on exit, we accounted for the infectivity profile specific to the variant upon exit from quarantine where cases that exhibited symptoms or tested positive isolated (1–3). To determine the probability of PQT, the estimated number of secondary cases were specified to be negative-binomially distributed (2, 3).

### Serial testing

To determine the extent of transmission for rapid antigen serial testing with a specified testing frequency and uniform risk of infection between tests, we calculated the number of secondary infections based on the infectivity profile for the variant in the context of individual and population vaccination, while incorporating isolation of cases that test positive or exhibit symptoms (3).

### Vaccination status

We stratified the population into unvaccinated, fully vaccinated (with only two-doses of the vaccine), and boosted (i.e. fully vaccinated with a booster dose). In the fully vaccinated group, the effectiveness of the vaccine is parameterized by values based on a 6-month or longer period since receiving the second dose. The boosted group refers to those who have received a third dose of vaccine at least 6 months after receiving the second dose.

## Supplementary Material

pgac100_Supplemental_File

## Data Availability

The computational code can be found at the online GitHub repository (21), and all relevant data is publicly available and cited in the main text or supplementary material.

## References

[1] Wells CR , et al. 2022. Quarantine and testing strategies to ameliorate transmission due to travel during the COVID-19 pandemic: a modelling study. Lancet Reg Health—Eur. 14:100304. 35036981 10.1016/j.lanepe.2021.100304 PMC8743228

[2] Wells CR , et al. 2021. Optimal COVID-19 quarantine and testing strategies. Nat Commun. 12:356. 33414470 10.1038/s41467-020-20742-8 PMC7788536

[3] Wells CR , et al. 2022. Comparative analyses of FDA EUA-approved rapid antigen tests and RT-PCR for COVID-19 quarantine and surveillance-based isolation. medRxiv. https://www.medrxiv.org/content/10.1101/2021.08.23.21262499v4, (accessed 2022 Feb 15). 2021.08.23.21262499 10.1038/s43856-022-00147-y PMC9271059 35822105

[4] Aleta A , et al. 2020. Modelling the impact of testing, contact tracing and household quarantine on second waves of COVID-19. Nat Hum Behav. 4:964–971. 32759985 10.1038/s41562-020-0931-9 PMC7641501

[5] Walensky RP , Walke HT, Fauci AS. 2021. SARS-CoV-2 variants of concern in the United States-challenges and opportunities. JAMA. 325:1037–1038. 33595644 10.1001/jama.2021.2294 PMC9009864

[6] Haque A , Pant AB. 2022. Mitigating Covid-19 in the face of emerging virus variants, breakthrough infections and vaccine hesitancy. J Autoimmun. 127:102792. 34995958 10.1016/j.jaut.2021.102792 PMC8719928

[7] Centers for Disease Control and Prevention . 2022. CDC Updates and Shortens Recommended Isolation and Quarantine Period for General Population. CDC Newsroom. https://www.cdc.gov/media/releases/2021/s1227-isolation-quarantine-guidance.html, (accessed 2022 Feb 2).

[8] CDC. 2022. Quarantine and Isolation. Centers for Disease Control and Prevention, https://www.cdc.gov/coronavirus/2019-ncov/your-health/quarantine-isolation.html, (accessed 2021 Jul 19).

[9] Ministère de L'europe et des Affaires étrangères (France Diplomacy—Ministry for Europe and Foreign Affairs) Isolation, testing, attending events—What are the rules in France?. Press Room, https://www.diplomatie.gouv.fr/en/coming-to-france/coming-to-france-your-covid-19-questions-answered/isolation-testing-attending-events-what-are-the-rules-in-france/, (updated 2022 Feb 17).

[10] E Redden 2020; Disparities in Testing, https://www.insidehighered.com/news/2020/08/21/covid-19-testing-strategies-vary-widely-across-institutions, (accessed 2022 Feb 21).

[11] National Health Service UK How long to self-isolate. https://www.nhs.uk/conditions/coronavirus-covid-19/self-isolation-and-treatment/how-long-to-self-isolate/, (accessed 2022 Feb 21).

[12] CDC. 2022. Testing for SARS-CoV-2 in non-healthcare workplaces. Centers for Disease Control and Prevention, https://www.cdc.gov/coronavirus/2019-ncov/community/organizations/testing-non-healthcare-workplaces.html, (accessed 2022 May 5).

[13] CDC. 2022. Overview of Testing for SARS-CoV-2, the virus that causes COVID-19. Centers for Disease Control and Prevention, https://www.cdc.gov/coronavirus/2019-ncov/hcp/testing-overview.html, accessed 2022 May 5.

[14] Kissler SM , et al. 2021. Viral dynamics of SARS-CoV-2 variants in vaccinated and unvaccinated persons. N Engl J Med. 385:2489–2491. 34941024 10.1056/NEJMc2102507 PMC8693673

[15] CDC. 2020. COVID data tracker. Centers for Disease Control and Prevention, https://covid.cdc.gov/covid-data-tracker, (accessed 2022 Feb 16).

[16] Ferretti L , et al. 2020. The timing of COVID-19 transmission. medRxiv. https://www.medrxiv.org/content/10.1101/2020.09.04.20188516v2, (accessed 2022 Feb 15).

[17] Hay JA , et al. 2022. Viral dynamics and duration of PCR positivity of the SARS-CoV-2 Omicron variant. medRxiv. https://www.medrxiv.org/content/10.1101/2022.01.13.22269257v2, (accessed 2022 April 28).

[18] Hellewell J , et al. 2021. Estimating the effectiveness of routine asymptomatic PCR testing at different frequencies for the detection of SARS-CoV-2 infections. BMC Med. 19:106. 33902581 10.1186/s12916-021-01982-x PMC8075718

[19] Gremmels H , et al. 2021. Real-life validation of the Panbio^TM^ COVID-19 antigen rapid test (Abbott) in community-dwelling subjects with symptoms of potential SARS-CoV-2 infection. EClinicalMedicine. 31:100677. 33521610 10.1016/j.eclinm.2020.100677 PMC7832943

[20] Linares M , et al. 2020. Panbio antigen rapid test is reliable to diagnose SARS-CoV-2 infection in the first 7 days after the onset of symptoms. J Clin Virol. 133:104659. 33160179 10.1016/j.jcv.2020.104659 PMC7561603

[21] Wells CR , et al. 2022. MATLAB Code: quarantine and serial testing for variants of SARS-CoV-2 with benefits of vaccination and boosting on consequent control of COVID-19. https://github.com/WellsRC/Vaccine_VOC_Quarantine. GitHub. (accessed 2022 May 25). 10.1093/pnasnexus/pgac100 PMC9335027 35909795

