# Supplementary material for: Quarantine and serial testing for variants of SARS-CoV-2 with benefits of vaccination and boosting on consequent control of COVID-19
Source: PNAS Nexus. 2022 Jul 27;1(3):pgac100. doi: 10.1093/pnasnexus/pgac100 (PMC9335027; doi:10.1093/pnasnexus/pgac100)
Supplement: pgac100_Supplemental_File [file pgac100_supplemental_file.pdf]

Supplementary Material: Quarantine and serial testing for variants of SARS-CoV-2 with benefits of vaccination and boosting on consequent control of COVID-19

Chad R. Wells[1], Abhishek Pandey[1], Senay Gokcebel[2,3], Gary Krieger[4,5], A. Michael Donoghue[6], Burton H. Singer[7], Seyed M. Moghadas[8], Alison P. Galvani[1], and Jeffrey P. Townsend [9, 10, 11]*

[1] Center for Infectious Disease Modeling and Analysis (CIDMA), Yale School of Public Health, New Haven, Connecticut 06520, USA
[2] Yale School of Public Health, New Haven, Connecticut 06510, USA
[3] Grinnell College, Grinnell, Iowa 50112, USA
[4] NewFields E&E, Boulder, Colorado 80301, USA.
[5] Skaggs School of Pharmacy and Pharmaceutical Science, University of Colorado Anschutz Medical Campus, Aurora, CO 80045, USA.
[6] Group HSE, BHP Group Ltd, 171 Collins Street, Melbourne, Victoria 3000, Australia.
[7] Emerging Pathogens Institute, University of Florida, P.O. Box 100009, Gainesville, Florida 32610, USA
[8] Agent-Based Modelling Laboratory, York University, Toronto, Ontario, Canada
[9] Department of Biostatistics, Yale School of Public Health, New Haven, Connecticut 06510, USA
[10] Program in Computational Biology and Bioinformatics, Yale University, New Haven, Connecticut 06511, USA
[11] Program in Microbiology, Yale University, New Haven, Connecticut 06511, USA

*Corresponding author: jeffrey.townsend@yale.edu

Transformation of infectivity

To transform the infectivity profile generated from data on the original SARS CoV-2 strain without vaccination to that of a variant of concern and/or vaccinated individual, we constructed a mapping between the Ct value and infectivity.

To perform this mapping from the infectivity of an unvaccinated case to the infectivity of a vaccinated case, we utilized the calculated slope at which the Ct value declines to its minimum and the slope at which the Ct value increases to 40 from Kissler et al (1) and Hay et al (2). A Ct value of 40 has been clinically specified as the upper bound of the Ct value in the detection of SARS CoV-2 infection in an individual (3, 4). For the purpose of the mapping, we extrapolated the Ct value past this upper bound of 40. The slope prior to the minimum Ct value in an unvaccinated individual was $m_N = (40 - 20.7) / (-3.5 - 0) = -6.0$. The slope before the minimum Ct value in a vaccinated individual was $m_V = (40 - 20.5) / (-3.2 - 0) = -6.1$. The rates at which the Ct value increased after the nadir in unvaccinated and vaccinated individuals were $m_N = (20.7 - 40) / (0 - 7.5) = 2.6$ and $m_V = (20.5 - 40) / (0 - 5.5) = 3.5$, respectively.

Let the infectivity of an unvaccinated individual infected with the original strain at time *t* in the disease time course be $r(t)$. Because the nadir values of the Ct could not be shown to be statistically significantly different between unvaccinated and vaccinated individuals who acquired infection (1), we set them to be equal. Accordingly, the infectivity of the vaccinated individual at time t is

$$r\left(m_V(t - t_P)/m_N + t_P\right),$$

where t_P is the time at which infectivity peaks.

For the Alpha and Delta variants of concern, we used a similar process. We set the Ct nadir values to be equal. The corresponding slopes prior to the minimum Ct values for the original strain, Alpha, and Delta variants were

$m_o = (40 - 20.1)/(-4.2 - 0) = -4.7,$

$m_{\alpha} = (40 - 21.0)/(-3.4 - 0) = -5.6$, and

$m_{\delta} = (40 - 19.8)/(-3.0 - 0) = -6.7$, respectively.

The slopes preceding the Ct nadir values for the original strain, Alpha, and Delta variants were

$m_{o} = (20.1 - 40)/(0 - 7.3) = 2.7$,

$m_{\alpha} = (21.0 - 40)/(0 - 6.2) = 3.1$, and

$m_{\delta} = (19.8 - 40)/(0 - 6.2) = 3.3$, respectively.

Thus, denoting infectivity in the unvaccinated with subscript *u* and the vaccinated with subscript *v*, as well as a second subscript α for the Alpha variant and subscript δ for the Delta variant,

$$r_{u,\alpha}(t) = r\left(m_{\alpha}(t - t_{P})/m_{o} + t_{P}\right),$$

$$r_{u,\delta}(t) = r\left(m_{\delta}(t - t_{P})/m_{o} + t_{P}\right),$$

$$r_{v,\alpha}(t) = r\left(\frac{m_{V}m_{\alpha}(t-t_{P})}{m_{N}m_{o}} + t_{P}\right), \text{ and}$$

$$r_{v,\delta}(t) = r\left(\frac{m_{V}m_{\delta}(t-t_{P})}{m_{N}m_{o}} + t_{P}\right).$$

Because there was no direct comparison of the Ct value for the original pandemic virus strain(s) and Omicron, a double transformation was required for Omicron. We first conducted the transformation from the original strain to the Delta variant in the absence of vaccination (i.e., $r_{u,\delta}(t) = r\left(m_{\delta}(t - t_{P})/m_{o} + t_{P}\right)$). The corresponding slope prior to the minimum Ct value for the Delta variant observed in the Omicron comparative study from Hay et al (2) was

$\bar{m}_{\delta} = (40 - 20.5)/(-4.67 - 0) = -4.2$.

The slope prior to the minimum Ct value for Omicron was

$\bar{m}_{o} = (40 - 23.3)/(-4.67 - 0) = -3.6$.

The corresponding slopes preceding the Ct nadir values for the Delta and Omicron variants were

$\bar{m}_{\delta} = (20.5 - 40)/(0 - 6.23) = 3.1$ and

$\bar{m}_o = (23.3 - 40)/(0 - 5.35) = 3.1$, respectively (2).

In a comparative study (2), the Ct nadir for Omicron was demonstrated to be slightly higher than that of Delta. However, an alternative study has highlighted that Omicron can exhibit more frequent low Ct values compared to Delta (5). A third study found that the two variants had similar infectious viral loads (6). To be consistent with our analyses of other variants of concern, we assumed that the Ct nadir for Delta and Omicron were equal. Thus, Ct slopes from Hay et al. (2) were used to provide parameters for calculations of

$$r_{u,o}(t) = r_{u,\delta}\left(\bar{m}_o(t - t_P)/\bar{m}_\delta + t_P\right) \text{ and } r_{v,o}(t) = r_{u,\delta}\left(\frac{m_V\bar{m}_o(t-t_P)}{m_N\bar{m}_\delta} + t_P\right).$$

Applying this transformation and assuming equal incubation periods, the infectivity of Omicron rises earlier than Delta after infection. In our analysis, this rapid rise in infectivity also corresponds to a faster increase in diagnostic sensitivity, which leads to a higher probability of detection of Omicron infection relative to Delta, consistent with an empirical comparative study conducted within households (5).

After the transformation of the infectivity profile from an unvaccinated individual infected with the original strain to that of the respective variant of concern, we re-normalized the infectivity such that it would generate the number of secondary infections expected in the absence of isolation and vaccination (7–9). For each variant, a distinct normalization constant in the absence of isolation and vaccination was also applied to generate the infectivity profile for a vaccinated individual.

To validate this transformation of the infectivity profile for each variant and vaccine status, we computed the R^2 value (i.e., squared Pearson coefficient) between the empirical data of Ct values and the infectivity profiles.

In the Kissler et al (1) study, they stratified infections as breakthrough or non-breakthrough infections, and based on the viral lineage (i.e., non-variant, Alpha, Delta, Epsilon, and Other). Specifying no vaccination, the R^2 value is 0.692, 0.662, and 0.755 for the original SARS CoV-2 strain,

the Alpha variant, and the Delta variant, respectively. Specifying vaccination, the R^2 value is 0.832, 0.77, and 0.724 for the original SARS CoV-2 strain, the Alpha variant, and the Delta variant, respectively.

The data from Hay et al (2) did not specify vaccination status and explicitly specified the infections with the Delta variant or Omicron variant (i.e., Omicron and suspected Omicron). Infections that were not Delta variant or Omicron variant were specified as other in the Hay et al (2) study. For the construction of the infectivity profile in the absence of vaccination, the R^2 value is 0.653, 0.48, 0.493, and 0.485 respectively for the Delta variant, confirmed Omicron, suspected Omicron, and confirmed or suspected Omicron. For the construction of the infectivity profile under the scenario of a vaccinated individual, the R^2 value is 0.626, 0.489, 0.501, and 0.493 respectively for the Delta variant, confirmed Omicron, suspected Omicron, and confirmed or suspected Omicron.

Mapping for diagnostic sensitivity

For a benchmark temporal RT-PCR diagnostic sensitivity curve, we fitted a log-Normal distribution to serial testing data from Hellewell et al (10) via maximum likelihood approach (11) applied to the distribution of incubation periods determined by Ashcroft et al (12). To construct the diagnostic sensitivity curve, we considered an incubation period of 5.72 days, based on the average of the distribution (12). This diagnostic sensitivity curve, $s(t)$, provides the probability that an individual infected with the original SARS CoV-2 strain has a positive RT-PCR test at time t post-infection. To construct the mapping from infectivity to diagnostic sensitivity, we used the relative infectivity of variant v,

$$\bar{r}_v(t) = r_v(t) / \max_t \{r_v(t)\},$$

where $r_v(t)$ is the infectivity curve for variant v. Using the benchmark diagnostic sensitivity and relative infectivity for the incubation period of 5.72 days of the original strain, we constructed two Cartesian mappings of the relative infectivity and diagnostic sensitivity, i.e., $(\bar{r}_V(t),\ s(t))$, stratified by

time before (i.e., $t \leq t_p$) and after (i.e., $t > t_p$) the peak of infectiousness. Thus, the RT-PCR diagnostic sensitivity for variant v was obtained through the piecewise mapping function $s_v(r_v(t))$.

We fitted a linear logit model to data of the percent positive agreement between Abbott Panbio and RT-PCR tests post-symptom onset (13, 14) using a maximum likelihood approach (11, 15). We then constructed a mapping using the relative infectivity (assuming variant-specific incubation periods but no transformation of infectivity profile from the original SARS CoV-2 strain) and the percent positive agreement. Since the percent positive agreement was only available after the appearance of symptoms, the mapping is based on the time following the peak of infectiousness (11, 15).

Model parameters

To quantify PQT and the effective reproduction number during serial testing of the original SARS CoV-2 strain and three variants, we required quantification of the incubation period, the basic reproduction number, the proportion of infections that are asymptomatic, the effectiveness of the vaccine in preventing symptomatic disease, and the effectiveness of the vaccine in preventing infection for each variant.

Estimated mean duration of the incubation period for the original strain is 6.3 days, Alpha is 5.0 days, Delta is 4.3 days, and Omicron is 3.2 days (16–21). Based on estimated reproduction numbers and relative infectiousness to other variants, the basic reproduction number for the original strain is 2.79, Alpha is 4.19, Delta is 5.08, and Omicron is 6.57 (7–9, 22).

In the absence of vaccination, the proportion of infections that are asymptomatic is 35.1% for the original strain and Alpha variant, 22.04% for Delta, and 27.5% for Omicron (23–26). The Alpha variant was specified to have equal probability of asymptomatic infection as the original strain, as the proportion of asymptomatic infections (11/51) in the control group for Alpha infection can not be ruled as significantly different than the proportion of asymptomatic infections (26/106) in the control group for non-Alpha infections (Fisher Exact test P = 0.8411) (24). The proportion of Delta infections that are

asymptomatic was calculated based on the reduction in the proportion of asymptomatic infections in unvaccinated individuals relative to the Alpha variant (25).

To determine the proportion of infections that are asymptomatic in vaccinated individuals, we use the specified proportion of infections that are asymptomatic in unvaccinated individuals and the effectiveness of vaccination in reducing symptomatic infection. The effectiveness of two doses of vaccine against symptomatic infection in Delta is 62.7%, and 8.8% for Omicron (27, 28). These estimates for the effectiveness of two doses are based on having received the second dose of the vaccine at least six months ago. There was an absence of information about the effectiveness of two doses of vaccine against symptomatic infection for the original SARS CoV-2 strain and Alpha based on having received the second dose of the vaccine at least six months ago. To estimate the effectiveness of the vaccine six-months after being fully vaccinated, we used an estimate from a meta-analysis which computed a 22.2 decrease in percentage points from one month to six months after being fully vaccinated (29). Specifying a 94% and 93% initial effectiveness against symptomatic infection from the original strain and Alpha respectively (30, 31), we estimate the effectiveness against symptomatic infection to be 71.8% for the original SARS CoV-2 strain and 70.8% for the Alpha variant of concern six months after being fully vaccinated. Thus, the proportion of asymptomatic infections for an individual vaccinated with two doses for the original strain is 81.7%, Alpha is 81.05%, Delta is 70.92%, and Omicron is 33.88%.

The effectiveness for a booster dose against symptomatic infection for Delta is 93.5%, and 67.3% for Omicron (27, 28). A booster dose was defined as the third dose of vaccine at least six months after receiving the second dose of vaccine in fully vaccinated individuals (27). We estimated the effectiveness for a booster dose against symptomatic infection in the original strain and the Alpha variant. Using the effectiveness of the second and third doses for the Delta variant, we determined the rate at which an additional dose improved effectiveness, denoted by ρ, from

$$\varepsilon_B = \varepsilon_2 + (1 - \varepsilon_2)\left(1 - e^{-\rho}\right),$$

where ε_B is the effectiveness of the booster and ε_2 is the effectiveness after receiving the second dose. From this equation and corresponding effectiveness of two vaccine doses for the original strain and alpha variant, we approximated the effectiveness for the booster dose. Thus, the proportion of breakthrough infections for individuals vaccinated with a booster dose that remain asymptomatic for the original strain is 96.81%, infected by Alpha is 96.70%, infected by Delta is 94.93%, and infected by Omicron is 76.29%.

The effectiveness of two-dose vaccination in preventing infection (i.e., diagnostic disease) is 63.7% for Delta, and 13.8% for Omicron (32). Similar to determining the reduction in effectiveness of two-dose vaccination in preventing symptomatic disease from the original SARS CoV-2 strain and the Alpha variant, we used the meta-analysis estimate of 18 percentage point reduction in effectiveness from one month to six months after vaccination. Specifying an initial effectiveness of 92% for the original SARS CoV-2 strain and 91.7% for the Alpha variant (31, 33–35), the effectiveness of two-dose vaccination in preventing infection six months after being fully vaccinated is 74% and 73.7%, respectively for the original SARS CoV-2 strain and the Alpha variant. The effectiveness of boosters in preventing infection is 94.2% for Delta, and 72.1% for Omicron (32). Using a similar approach described above, we estimated the effectiveness of the booster in preventing infection from the original strain and the Alpha variant to be 95.85% and 95.80%, respectively.

For a specified quarantine or serial testing strategy, we denote the amount of transmission from an unvaccinated individual by R_N, an individual vaccinated with two doses by R_V, and transmission from an individual receiving a booster by R_B. To determine these amounts of transmission, we used the framework presented by Wells et al (11). This framework considers a random entry into quarantine over the period of asymptomatic infection (i.e., the incubation period for symptomatic cases and the duration of disease for asymptomatic) and isolation upon symptom onset or a positive test. For those not identified in quarantine, the extent of remaining transmission is reduced based on the duration of quarantine q. In the context of serial testing, infected individuals isolate upon symptom onset or a

positive test, with transmission occurring between tests with a frequency of every *f* days. Specifying v_2 as the vaccine uptake of two doses and $\varepsilon_{I,2}$ as the effectiveness of two vaccine doses in preventing infection, the number of secondary cases following a quarantine of q days was quantified as

$$\frac{(1-v_2)}{(1-v_2)+v_2(1-\varepsilon_{I,2})}R_N(q)(1-v_2\varepsilon_{I,2})+\frac{v_2(1-\varepsilon_{I,2})}{(1-v_2)+v_2(1-\varepsilon_{I,2})}R_V(q)(1-v_2\varepsilon_{I,2}).$$

With 100% of the population receiving two doses of vaccines, denoting the booster uptake by v_B, and the effectiveness of boosters in preventing infection by $\varepsilon_{I,B}$, the number of secondary cases following a quarantine of q days was quantified by

$$\frac{(1-v_B)(1-\varepsilon_{I,2})}{(1-v_B)(1-\varepsilon_{I,2})+v_B(1-\varepsilon_{I,B})}R_V(q)\left(1-\left[(1-v_B)\varepsilon_{I,2}+v_B\varepsilon_{I,B}\right]\right)+\frac{v_B(1-\varepsilon_{I,B})}{(1-v_B)(1-\varepsilon_{I,2})+v_B(1-\varepsilon_{I,B})}R_B(q)\left(1-\left[(1-v_B)\varepsilon_{I,2}+v_B\varepsilon_{I,B}\right]\right).$$

Similarly, specifying v_2 as the vaccine uptake of two doses and $\varepsilon_{I,2}$ as the effectiveness of two vaccine doses in preventing infection, the number of secondary cases with testing frequency of every *f* days was quantified by

$$\frac{(1-v_2)}{(1-v_2)+v_2(1-\varepsilon_{I,2})}R_N(f)(1-v_2\varepsilon_{I,2})+\frac{v_2(1-\varepsilon_{I,2})}{(1-v_2)+v_2(1-\varepsilon_{I,2})}R_V(f)(1-v_2\varepsilon_{I,2}).$$

In addition, with 100% of the population receiving two doses of vaccines, v_B as the booster uptake, and $\varepsilon_{I,B}$ as the effectiveness of boosters in preventing infection, the number of secondary cases with testing frequency of every *f* days was quantified as

$$\frac{(1-v_B)(1-\varepsilon_{I,2})}{(1-v_B)(1-\varepsilon_{I,2})+v_B(1-\varepsilon_{I,B})}R_V(f)\left(1-\left[(1-v_B)\varepsilon_{I,2}+v_B\varepsilon_{I,B}\right]\right)+\frac{v_B(1-\varepsilon_{I,B})}{(1-v_B)(1-\varepsilon_{I,2})+v_B(1-\varepsilon_{I,B})}R_B(f)\left(1-\left[(1-v_B)\varepsilon_{I,2}+v_B\varepsilon_{I,B}\right]\right).$$

Supplementary References Cited

1. S. M. Kissler, *et al.*, Viral Dynamics of SARS-CoV-2 Variants in Vaccinated and Unvaccinated Persons. *New England Journal of Medicine* **385**, 2489–2491 (2021).

2. J. A. Hay, *et al.*, Viral dynamics and duration of PCR positivity of the SARS-CoV-2 Omicron variant. *medRxiv*, 2022.01.13.22269257 (2022).

3. , "Understanding cycle threshold (Ct) in SARS-CoV-2 RT-PCR: A guide for health protection teams" (Public Health England, 2020) (April 27, 2022).

4. C. Johnston, B. Healy, Interpretation of COVID-19 PCR testing- what surgeons need to know. *Br. J. Surg.* **107**, e367 (2020).

5. F. P. Lyngse, *et al.*, SARS-CoV-2 Omicron VOC Transmission in Danish Households. *medRxiv*, 2021.12.27.21268278 (2021).

6. O. Puhach, *et al.*, Infectious viral load in unvaccinated and vaccinated patients infected with SARS-CoV-2 WT, Delta and Omicron. *medRxiv*, 2022.01.10.22269010 (2022).

7. Y. Liu, J. Rocklöv, The reproductive number of the Delta variant of SARS-CoV-2 is far higher compared to the ancestral SARS-CoV-2 virus. *Journal of Travel Medicine* **28** (2021).

8. W. Yang, J. Shaman, SARS-CoV-2 transmission dynamics in South Africa and epidemiological characteristics of the Omicron variant. *medRxiv* (2021) https:/doi.org/10.1101/2021.12.19.21268073.

9. K. Tao, *et al.*, The biological and clinical significance of emerging SARS-CoV-2 variants. *Nat. Rev. Genet.* **22**, 757–773 (2021).

10. J. Hellewell, *et al.*, Estimating the effectiveness of routine asymptomatic PCR testing at different frequencies for the detection of SARS-CoV-2 infections. *BMC Med.* **19**, 106 (2021).

11. C. R. Wells, *et al.*, Comparative analyses of FDA EUA-approved rapid antigen tests and RT-PCR for COVID-19 quarantine and surveillance-based isolation. *medRxiv*, 2021.08.23.21262499 (2022).

12. P. Ashcroft, S. Lehtinen, D. C. Angst, N. Low, S. Bonhoeffer, Quantifying the impact of quarantine duration on COVID-19 transmission. *Elife* **10** (2021).

13. H. Gremmels, *et al.*, Real-life validation of the Panbio™ COVID-19 antigen rapid test (Abbott) in community-dwelling subjects with symptoms of potential SARS-CoV-2 infection. *EClinicalMedicine* **31**, 100677 (2021).

14. M. Linares, *et al.*, Panbio antigen rapid test is reliable to diagnose SARS-CoV-2 infection in the first 7 days after the onset of symptoms. *J. Clin. Virol.* **133**, 104659 (2020).

15. C. R. Wells, *et al.*, Quarantine and testing strategies to ameliorate transmission due to travel during the COVID-19 pandemic: a modelling study. *Lancet Reg Health Eur* **14**, 100304 (2022).

16. H. Xin, *et al.*, The Incubation Period Distribution of Coronavirus Disease 2019: A Systematic Review and Meta-analysis. *Clin. Infect. Dis.* **73**, 2344–2352 (2021).

17. R. Grant, *et al.*, Impact of SARS-CoV-2 Delta variant on incubation, transmission settings and vaccine effectiveness: Results from a nationwide case-control study in France. *Lancet Reg Health Eur* **13**, 100278 (2022).

18. L. Jansen, *et al.*, Investigation of a SARS-CoV-2 B.1.1.529 (Omicron) Variant Cluster - Nebraska,

November-December 2021. *MMWR Morb. Mortal. Wkly. Rep.* **70**, 1782–1784 (2021).

19. L. T. Brandal, *et al.*, Outbreak caused by the SARS-CoV-2 Omicron variant in Norway, November to December 2021. *Euro Surveill.* **26** (2021).

20. J. A. Backer, *et al.*, Shorter serial intervals in SARS-CoV-2 cases with Omicron BA.1 variant compared with Delta variant, the Netherlands, 13 to 26 December 2021. *Eurosurveillance* **27** (2022).

21. G. Helmsdal, *et al.*, Omicron Outbreak at a Private Gathering in the Faroe Islands, Infecting 21 of 33 Triple-Vaccinated Healthcare Workers. *Clinical Infectious Diseases* (2022) https:/doi.org/10.1093/cid/ciac089.

22. S. B. Jørgensen, K. Nygård, O. Kacelnik, K. Telle, Secondary Attack Rates for Omicron and Delta Variants of SARS-CoV-2 in Norwegian Households. *JAMA* **327**, 1610–1611 (2022).

23. P. Sah, *et al.*, Asymptomatic SARS-CoV-2 infection: A systematic review and meta-analysis. *Proc. Natl. Acad. Sci. U. S. A.* **118** (2021).

24. K. R. W. Emary, *et al.*, Efficacy of ChAdOx1 nCoV-19 (AZD1222) vaccine against SARS-CoV-2 variant of concern 202012/01 (B.1.1.7): an exploratory analysis of a randomised controlled trial. *Lancet* **397**, 1351–1362 (2021).

25. D. Loconsole, *et al.*, Changing Features of COVID-19: Characteristics of Infections with the SARS-CoV-2 Delta (B.1.617.2) and Alpha (B.1.1.7) Variants in Southern Italy. *Vaccines* **9**, 1354 (2021).

26. J. J. Lee, *et al.*, Importation and Transmission of SARS-CoV-2 B.1.1.529 (Omicron) Variant of Concern in Korea, November 2021. *J. Korean Med. Sci.* **36**, e346 (2021).

27. E. K. Accorsi, *et al.*, Association Between 3 Doses of mRNA COVID-19 Vaccine and Symptomatic Infection Caused by the SARS-CoV-2 Omicron and Delta Variants. *JAMA* **327**, 639–651 (2022).

28. N. Andrews, *et al.*, Covid-19 Vaccine Effectiveness against the Omicron (B.1.1.529) Variant. *N. Engl. J. Med.* **386**, 1532–1546 (2022).

29. D. R. Feikin, *et al.*, Duration of effectiveness of vaccines against SARS-CoV-2 infection and COVID-19 disease: results of a systematic review and meta-regression. *Lancet* **399**, 924–944 (2022).

30. A. Vitiello, F. Ferrara, V. Troiano, R. La Porta, COVID-19 vaccines and decreased transmission of SARS-CoV-2. *Inflammopharmacology* **29**, 1357–1360 (2021).

31. N. Dagan, *et al.*, BNT162b2 mRNA Covid-19 Vaccine in a Nationwide Mass Vaccination Setting. *N. Engl. J. Med.* **384**, 1412–1423 (2021).

32. H. F. Tseng, *et al.*, Effectiveness of mRNA-1273 against SARS-CoV-2 Omicron and Delta variants. *Nat. Med.* (2022) https:/doi.org/10.1038/s41591-022-01753-y.

33. E. S. Rosenberg, *et al.*, New COVID-19 Cases and Hospitalizations Among Adults, by Vaccination Status - New York, May 3-July 25, 2021. *MMWR Morb. Mortal. Wkly. Rep.* **70**, 1306–1311 (2021).

34. T. Harder, *et al.*, Effectiveness of COVID-19 vaccines against SARS-CoV-2 infection with the Delta (B.1.617.2) variant: second interim results of a living systematic review and meta-analysis, 1 January to 25 August 2021. *Eurosurveillance* **26**, 2100920 (2021).

35. M. G. Thompson, *et al.*, Effectiveness of a Third Dose of mRNA Vaccines Against COVID-19–Associated Emergency Department and Urgent Care Encounters and Hospitalizations Among Adults During Periods of Delta and Omicron Variant Predominance — VISION Network, 10 States, August 2021–January 2022. *MMWR. Morbidity and Mortality Weekly Report* **71**, 139–145 (2022).
